# The quantile time–frequency connectedness of economic policy uncertainty between China and the G7 countries

**DOI:** 10.1371/journal.pone.0337444

**Published:** 2025-12-01

**Authors:** Jizhi Zhao, Guangfu Chen, Ying Song

**Affiliations:** 1 School of Business, Wuyi University, Wuyishan, Fujian, China; 2 School of Mathematics and Computer, Wuyi University, Wuyishan, Fujian, China; Kean University, UNITED STATES OF AMERICA

## Abstract

Most of the existing studies on the connectedness among economic policy uncertainties (EPUs) usually neglect the quantile and frequency domain perspectives. To address this limitation, this paper proposes a quantile time–frequency connectedness model to analyze the connectedness among EPUs by combining the quantile and frequency domain dimensions. First, the quantile-vector autoregressive model (QVAR(p)) is estimated and converted into the quantile-vector moving average representation (QVMA(∞)). Next, the generalized prediction error variance decomposition (GFEVD) is computed, from which various types of time-domain connectedness metrics are calculated. Finally, the spectral decomposition method is used to compute frequency-domain connectedness metrics and establish a link between time- and frequency-domain metrics. The empirical results of this paper, based on the sample data of China and G7 countries, reveal several important findings. The EPU of the United States acts as a net transmitter of shocks in both the short and long term, whereas China functions as a net receiver of shocks. The total connectedness index (TCI) demonstrates significant heterogeneity, with its dynamics primarily driven by short-term rather than long-term components. Additionally, connectedness shows substantial improvement under extreme conditions.

## Introduction

Economic policy uncertainty (EPU), as a concentrated manifestation of the unpredictability of policy changes and their outcomes, has become an important causal factor of global economic volatility and financial market risk [1]. It arises from the ambiguity in the decision-making process of policymakers and the structural disturbances in the economic environment caused by external shocks. So, what kind of heterogeneity exists in the connectedness of EPUs across economies in terms of short-term fluctuations and long-term trends? How does the contagion path of EPUs between economies evolve dynamically under normal and extreme conditions? Addressing the above questions not only provides a dynamic perspective for the study of policy spillovers, but also helps international organizations to build a multilateral risk early warning mechanism to enhance the resilience of the global economic governance system, and at the same time helps investors to identify tail risk correlations and improve cross-market hedging strategies.

There are three ways to calculate EPU. First, it can be measured by the volatility of a single economic variable, such as the implied volatility of stock market returns or the volatility of forecast errors [2–6]. Second, it is measured using non-economic dummy variables such as wars, political scandals, changes in government, changes in officials, and terms in office [7–10]. Third, it is measured based on the frequency of news reports, such as the EPU index compiled by Baker et al. [1] and Huang and Luk [11]. In this paper, we choose the EPU index to measure the level of economic policy uncertainty. The EPU index has the advantages of traceability, continuity, and time variation, and can more accurately reflect the dynamic changes in economic policy uncertainty.

According to Baker et al. [1], the global EPU index rose sharply to a historical high of 460.18 (Global EPU Index data is available at https://www.policyuncertainty.com/global_monthly.html.) in January 2025. Looking at the evolution of the EPU index in recent years, the most striking feature is the sharp fluctuations. Both the magnitude and the frequency of the fluctuations have significantly exceeded historical norms, reflecting the growing challenge of economic policy uncertainty that the world is facing. It is not difficult to see that both domestic political dynamics, regional military conflicts and economic crises have invariably triggered EPU spillovers, which have further exacerbated the volatility of the EPU at the global level.

According to the existing literature, current academic research focuses on the impact of EPUs on macroeconomic variables within a single country and analyzes their transmission mechanism. However, EPUs can also generate connectedness effects through direct (e.g., trade, capital flows, etc.), indirect (e.g., similarity in economic policies, cultural background, etc.), and informational (e.g., investors’ psychological expectations, etc.) channels between economies, and some literatures have verified the existence of connectedness between EPUs across economies [12,13], but there is no in-depth analysis of the magnitude and direction of connectedness in the quantile and frequency domain dimensions.

Therefore, we use the method of Chatziantoniou et al. [14] to measure the dynamic connectedness and structural characteristics between China’s EPU and those of the G7 countries under different frequency domains (short-term and long-term) and different quantile levels (normal state and extreme state). The main differences with Chatziantoniou et al. [14] are as follows: First, the research perspectives are different. This paper focuses on the connectedness of EPU between economies, while the latter mainly studies the spillover effects between green bonds, green stocks and clean energy markets. Second, the research focus is different. This paper focuses on the measurement and formation mechanism of connectedness, while the latter mainly focuses on the construction of connectedness methods.

The contributions of this paper can be summarized as follows:

- Based on the quantile-frequency perspective, this paper constructs the connectedness network between China and G7 EPUs and explores the structural characteristics of their connectedness from both static and dynamic perspectives, comprehensively capturing the statistical characteristics of connectedness in the time dimension.
- By thoroughly exploring the magnitude and direction of EPU connectedness under different quantiles, new ideas are provided for the quantitative analysis of EPU connectedness.
- It is concluded that under extreme circumstances, the dynamic total connectedness between China and the EPU of the G7 countries is more significant.

The structure of this paper is organized as follows: Section **Literature review** systematically examines the existing scholarly works, Section **Methodology** introduces the empirical approaches adopted in this study, Section **Data and model parameter** Settings details the data sources and specifies the model parameter configurations. Subsequently, Section **Empirical results** will carry out the testing of research hypotheses through empirical analysis, and Section **Conclusion** will synthesize the key findings and implications of the study.

## Literature review

The existing literature on EPU spillovers can be divided into two main categories: The first type is research on the spillover effects of EPU on the macroeconomy and financial markets. Using econometric methods such as panel regression models and vector autoregression (VAR) models, the impact of EPU in one economy on output, consumption, investment, CPI, asset prices, and financial market risks in other economies has been studied [15–22].

The second type is the study of the measurement and performance of the EPU connectedness level, and the research in this paper belongs to this category. Most of this literature uses the spillover index method, which is based on the generalized error decomposition of the VAR model proposed by Diebold and Yılmaz [23], to measure the spillover effects of the EPU in different countries or economies.For example, Gabauer and Gupta [24] focused on analyzing the spillover effect of EPU between Japan and the United States. Their research found that trade shocks significantly increase the uncertainty spillover effect between the two countries. Klößner and Sekkel [25] investigated the spillover effect of EPU between the United States, Canada, the United Kingdom, France, Germany, Italy, and other developed countries. The results showed that this spillover effect was significant, even more than 50%. Since the outbreak of the subprime mortgage crisis, the United States and the United Kingdom have become important sources of spillovers, while other countries have mainly played the role of recipients of EPU during this period and later.Yin and Han [26] focused their research on the BRICS countries, and their empirical analysis showed that during the US subprime mortgage crisis, the spillover effects caused by the EPU in the BRICS countries reached their respective historical highs. Antonakakis et al. [27] investigated the potential spillover effects between the US, the EU, the UK, Japan, and Canada, and the results confirmed that the main source of spillovers to the EU came from the US.Tang et al. [28] found that there is a complex and closely related EPU network in the Asia-Pacific region, with both developed and developing economies as important network nodes, but the EPU spillover network is still dominated by a small number of developed economies. Bai et al. [29] found that the top six economies are strongly interconnected in terms of EPU, and most risk spillover effects are only observed at short frequencies of one to three months. The United States is an important transmitter of spillovers, while the United Kingdom and China are the main receivers of spillovers.

In summary, although scholars have made some progress in studying the connectedness of EPUs, most of the existing studies analyze the short- and long-term connectedness of EPUs in isolation, often ignoring their structural characteristics in different frequency domains and at different quantile levels. In view of this, this paper selects China and the G7 countries as research objects to explore in depth the connectedness between EPUs and its structural evolution in these eight economies since the 21st century.

## Methodology

This study adopts the quantile connectivity approach introduced by Chatziantoniou et al. [14] to explore the quantile propagation mechanism of economic policy uncertainty. Building on the connectivity analysis framework established by Diebold and Yılmaz [23], this methodology extends its applicability along an additional dimension: it enables the examination of connectivity across different frequencies for a given quantile, as well as the analysis of connectivity patterns across various quantiles for a specific frequency.

It should be noted that the TVP-VAR method developed by Antonakakis et al. [30] has also been widely employed in studying time-varying and frequency-specific connectivity [31–33]. However, compared to the TVP-VAR approach, the quantile time–frequency connectivity framework proposed by Chatziantoniou et al. [14] offers distinct advantages. Not only does it reveal connectivity features across time and frequency domains, but it also provides connectivity information at multiple quantile levels (e.g., 0.05, 0.15, ... , 0.95). This allows for a more comprehensive capture of the interdependence among variables under varying market conditions.

For a vector process $Y_{t}=(Y_{1t},Y_{2t},\cdots ,Y_{Nt}{)}^{\prime },t=1,2,\cdots ,T$ with *N* variables, its pth-order quantile vector autoregression model *QVAR*(*p*) is defined as follows:

$$Y_{t}=\mu_{t}(\tau )+\sum_{p=1}^{P}\beta_{p}(\tau )Y_{t-p}+\varepsilon_{t}(\tau ),\;\text{for }t=1,\cdots ,T$$ (1)

where *Y*_*t*_ and *Y*_*t*−1_,*Y*_*t*−2_,⋯,*Y*_*t*−*p*_ are *N* × 1 endogenous variables. *τ* represents the quantile number, and τ∈[0,1]. *p* denotes the lag order of the *QVAR* model. μ(τ) is an *N* × 1 dimensional intercept term. $\varepsilon_{t}(\tau )$ is an *N* × 1 dimensional random error term, and $\varepsilon_{t}(\tau )\sim i.i.d.(0,\Sigma (\tau ))$. According to Wold’s Theorem, if a *QVAR*(*p*) process satisfies the covariance stationarity condition, it can be represented as an infinite-order quantile vector moving average model, namely QVMA(∞):

$$Y_{t}=\mu (\tau )+\sum_{i=0}^{\infty }B_{i}(\tau )\varepsilon_{t-i}$$ (2)

In the generalized forecast error variance decomposition (GFEVD), the proportion *R*_*ij*,*H*_ of the variance of the H-step forecast error of variable *i* that is explained by variable *j*, i.e., the level of connectedness, is defined as follows:

$$R_{ij,H}=\frac{[\Sigma (\tau ){]}_{jj}^{-1}\sum_{h=0}^{H}[(B_{h}(\tau )\Sigma (\tau ){)}_{ij}{]}^{2}}{\sum_{h=0}^{H}[B_{h}(\tau )\Sigma (\tau )B_{h}^{\prime }(\tau ){]}_{ii}}$$ (3)

Eq (3) takes on the following form after standardization:

$${\tilde{R}}_{ij,H}=\frac{R_{ij,H}}{\sum_{k=1}^{N}R_{ij,H}}$$ (4)

${\tilde{R}}_{ij,H}$ measures the EPU connectedness between economy *i* and economy *j* under the H-step forecast period. If the quantile *τ* changes, it means that the shock state changes. Therefore, the EPU connectedness between economies can be calculated under different quantiles.

The total connectedness index (TCI), directional connectedness index (TO and FROM), net total directional connectedness (NET), and net pairwise directional connectedness (NPDC) defined at the quantile *τ* level are:

$$\left\{ \begin{array}{c} TO_{i\to j,H}=\sum_{\begin{array}{c} i=1,i\neq j \end{array}}^{N}{\tilde{R}}_{ji,H}\\ FROM_{i\leftarrow j,H}=\sum_{\begin{array}{c} i=1,i\neq j \end{array}}^{N}{\tilde{R}}_{ij,H}\\ TCI_{H}=N^{-1}\sum_{i=1}^{N}TO_{i,H}=N^{-1}\sum_{i=1}^{N}FROM_{i,H}\\ NET_{i,H}=TO_{i\to j,H}-FROM_{i\leftarrow j,H}\\ NPDC_{ij,H}={\tilde{R}}_{ij,H}-{\tilde{R}}_{ji,H} \end{array}\right.$$ (5)

where $TO_{i\to j,H}$ indicates the spillover from economy *i* to economy *j* and $FROM_{i\leftarrow j,H}$ indicates the spillover from economy *j* to economy *i*. TCI stands for the total connectedness index, which indicates the average impact of a series of shocks on all other shocks. The higher the TCI value, the stronger the EPU connectedness between economies and vice versa. *NPDC*_*ij*,*H*_ is the net pairwise connectedness index. When *NPDC*_*ij*,*H*_>0, series *i* is driven by series *j*, i.e., the impact of series *j* on series *i* is greater than the impact of series *j* on series *j*. *NET*_*i*,*H*_ stands for net directional connectedness index. A positive value indicates that economy *i* is a net sender in the EPU, while a negative value indicates that it is a net receiver.

Finally, consider the connectedness in the frequency domain, i.e., the level of connectedness of the EPU in different cycles (short and long term). The frequency domain GFEVD can be composed of two parts: spectral density and GFEVD. Similar to the time domain, the frequency GFEVD is normalized and the result is expressed as follows:

$$\left\{ \begin{array}{c} R_{ij}(\omega )=\frac{{\left[\sum_{h=0}^{\infty }(B(\tau )e^{-i\omega h}\Sigma (\tau ){)}_{ij}\right]}^{2}}{{\left[\Sigma (\tau )\right]}_{jj}^{-1}\sum_{h=0}^{\infty }[B(e^{-i\omega h})\Sigma (\tau )B(\tau )e^{i\omega h}{]}_{ii}}\\ {\tilde{R}}_{ij}(\omega )=\frac{R_{ij}(\omega )}{\sum_{k=1}^{N}R_{ij}(\omega )} \end{array}\right.$$ (6)

where ${\tilde{R}}_{ij}(\omega )$ is the spectral component of variable *i* at a given frequency due to the shock of variable *j*. We compute the short-run and long-run levels of EPU connectedness between economies by integrating over all frequencies within a given range (d), where d=(a,b):a,b∈(−π,π),a<b. The frequency connectedness ${\tilde{R}}_{ij}(\omega )$ is defined as follows:

$${\tilde{R}}_{ij,d}=\int_{a}^{b}{\tilde{R}}_{ij}(\omega )\;d\omega$$ (7)

Unlike ${\tilde{R}}_{ij,H}$, ${\tilde{R}}_{ij,d}$ reflects the connectedness of EPU between economies within a specific frequency range. Therefore, we define a set of connectedness within a specific frequency range *d* as follows:

$$\left\{ \begin{array}{c} TO_{i\to j,d}=\sum_{\begin{array}{c} i=1,i\neq j \end{array}}^{N}{\tilde{R}}_{ji,d}\\ FROM_{i\leftarrow j,d}=\sum_{\begin{array}{c} i=1,i\neq j \end{array}}^{N}{\tilde{R}}_{ij,d}\\ TCI(d)=N^{-1}\sum_{i=1}^{N}TO_{i\to j,d}=N^{-1}\sum_{i=1}^{N}FROM_{i\leftarrow j,d}\\ NET_{i,d}=TO_{i\to j,d}-FROM_{i\leftarrow j,d}\\ NPDC_{ij,d}={\tilde{R}}_{ij,d}-{\tilde{R}}_{ji,d} \end{array}\right.$$ (8)

Eq (8), which is interpreted in a similar way to Eq (5), provides information on the connectedness of EPU between economies in different frequency bands.

## Data and model parameter settings

### Data

In selecting the EPU data for this paper, we considered the availability and consistency of the data, as well as economies that have been closely linked to China’s economy and trade in recent years. The final sample countries are: China (CN) and the G7 countries (the United States, the United Kingdom, France, Italy, Germany, Canada, and Japan, abbreviated as US, UK, FR, IT, GE, CA, and JP, respectively). The sample period is from January 2000 to March 2025, with a frequency of monthly. The data for China is a smooth splicing of adjusted SCMP and Mainland Data. All of the above data are taken from the official website of “Economic Policy Uncertainty” (https://www.policyuncertainty.com/).

To ensure the stationarity of the EPU index series, this paper applies first-order differencing to the raw data. Drawing on the generation logic and economic connotations of EPU, we select the 0.05, 0.50, and 0.95 quantiles for analysis: The 0.05 quantile represents the “sharp decline period” of EPU, reflecting increased policy certainty; the 0.50 quantile represents the “normal fluctuation period” of EPU; and the 0.95 quantile represents the “sharp increase period” of EPU, reflecting a phase of high policy uncertainty.

Table 1 shows the descriptive statistics of the sample data. From the data, several key observations emerge. Canada exhibits the highest mean value (4.952) during the sample period, whereas Japan’s mean value is significantly lower than that of other economies (0.183). The standard deviation reflects the degree of fluctuation in EPU across the sample countries, with the United Kingdom, Germany, and China ranking highest, while Japan has the lowest standard deviation (23.327), indicating relatively stable EPU levels. In terms of skewness, France, Japan, and the United Kingdom display a left-skewed distribution, while the remaining economies exhibit a right-skewed distribution. Kurtosis measures reveal that all series follow a peaked distribution. The Jarque-Bera (JB) statistic confirms that none of the series are normally distributed at the 1% significance level. Additionally, the ERS unit root test results indicate stationarity in all series at the 1% significance level. Finally, each series demonstrates the presence of an ARCH effect.

**Table 1:** Descriptive statistics for the first-order difference series of EPU.

Variable	Mean	Std.Dev	Skewness	Kurtosis	JB	ERS	ARCH(8)-LM
*CN*	3.002	73.029	0.166	4.265	235.529***(0.000)	–4.987***(0.000)	113.102***(0.000)
*CA*	4.952	73.654	1.091	4.101	277.060***(0.000)	–2.709***(0.007)	27.594***(0.000)
*FR*	1.716	71.144	–0.315	2.044	59.326***(0.000)	–8.133***(0.000)	38.780***(0.000)
*GE*	3.257	75.323	0.727	3.810	213.944***(0.000)	–6.326***(0.000)	113.856***(0.000)
*IT*	0.499	36.273	0.126	1.582	33.506***(0.000)	–2.920***(0.004)	70.570***(0.000)
*JP*	0.183	23.327	–0.245	4.316	242.797***(0.000)	–11.406***(0.000)	137.596***(0.000)
*UK*	1.764	83.080	–1.334	17.611	4054.215***(0.000)	–8.425***(0.000)	22.134***(0.002)
*US*	1.182	45.843	0.290	4.260	237.801***(0.000)	–6.343***(0.000)	100.797***(0.000)

Note: ***, **,  denote significance at 1%, 5% and 10% significance level; JB represents the Jarque–Bera test statistics; ERS means ERS unit-root test statistics; ARCH (8)-LM means Lagrange Multiplier test statistic with a lag order of 8, and P-values for test statistics are indicated within parentheses.

**Fig 1:**
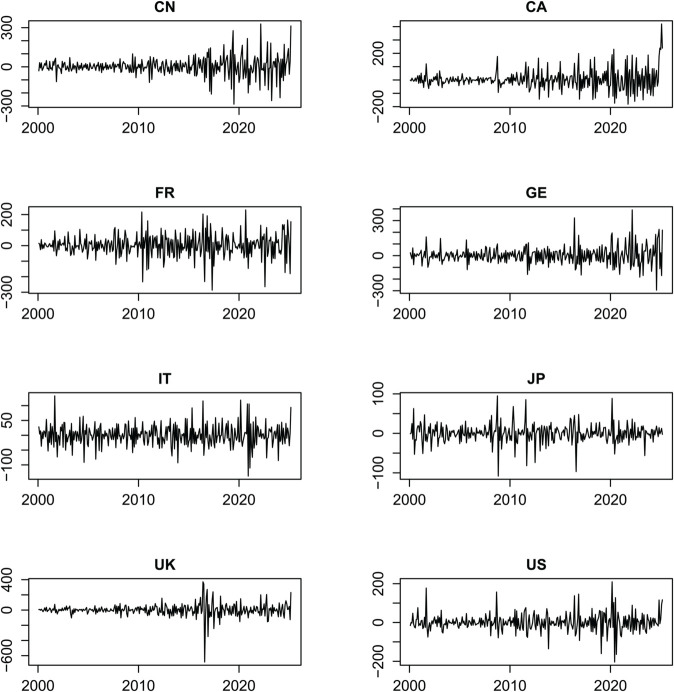
The first difference sequence of the EPU in China and the G7 countries.

Fig 1 shows the first-order difference sequence of China’s and the G7 countries’ EPU index during the sample period. From this sequence, we can see that the EPU of the sample countries has its own time-varying characteristics. China’s EPU fluctuates the most, followed by the United Kingdom and France. In addition, when international events such as the financial crisis in 2008, Brexit in 2016, and COVID-19 occur, the sample countries show some common characteristics.

From Table 2, we can see that the correlation coefficients between the EPUs of the sample countries range from 0.146 to 0.490, indicating that there is no strong correlation. For example, the correlation coefficient between China and Japan is 0.146, indicating that there is some correlation between the EPU of the two countries, but it is very weak; while the correlation coefficient between China and Germany is 0.384, indicating that there is a relatively strong correlation between the EPU of the two countries.

**Table 2:** Correlation Coefficients of EPU Between China and G7.

Variable	CN	CA	FR	GE	IT	JP	UK	US
*CN*	1.000							
*CA*	0.203	1.000						
*FR*	0.302	0.178	1.000					
*GE*	0.384	0.324	0.477	1.000				
*IT*	0.153	0.122	0.267	0.258	1.000			
*JP*	0.146	0.276	0.264	0.169	0.251	1.000		
*UK*	0.272	0.248	0.375	0.369	0.208	0.214	1.000	
*US*	0.324	0.468	0.365	0.490	0.250	0.316	0.323	1.000

### Model parameter settings

In this paper, the lag order of the frequency-domain quantile model is set to 1, as determined by the Bayesian Information Criterion (BIC). Meanwhile, the rolling window length is specified as 36 months—corresponding to an approximate 3-year observation period—with the primary objective of measuring the connectivity of EPU across sample countries. The selection of a 36-month rolling window is primarily driven by the volatility characteristics of EPU. Dynamic fluctuations in EPU are typically closely tied to macroeconomic cycles and major policy-related events (e.g., the 2008 Global Financial Crisis and the 2020 COVID-19 pandemic). The transmission and impact of such shocks are most pronounced over the medium term (roughly 2–4 years). A 36-month window effectively captures these medium-term fluctuations, while mitigating two key limitations: it avoids excessive noise introduction associated with shorter windows (e.g., 12 months) and prevents the smoothing of potential structural changes that may occur with longer windows (e.g., 60 months). Robustness check results are available upon request from the authors.

Similar to Chatziantoniou and Stenfors [34] and Chatziantoniou et al. [14], this paper divides the data into two different frequency bands to calculate the short- and long-term connectedness performance of EPU in the sample countries. $d_{1}=(\pi /5,\pi )$ is the high frequency band with a period length of 1 month to 5 months, representing the short term; $d_{2}=(0,\pi /5)$ is the low frequency band with a period length of more than 6 months, representing the long term.

In addition, in this paper, we use the ConnectednessApproach package [35] in R to calculate the relevant data results in Table 3 and to draw the visual plots in Figs 3, 4, 5, 6, and 7.

**Fig 2:**
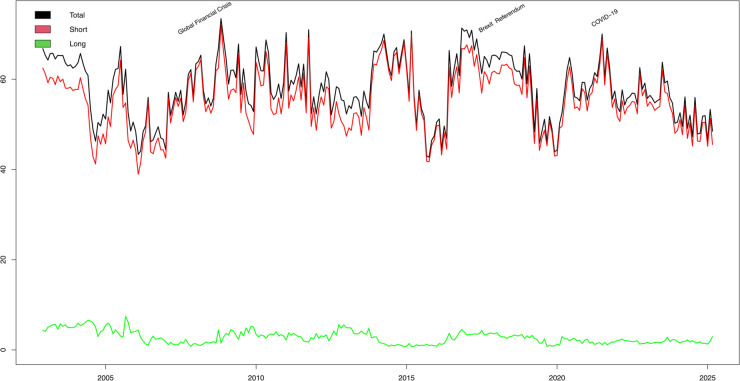
Dynamic Total Connectedness: Short-term, Long-term, and Overall.

**Fig 3:**
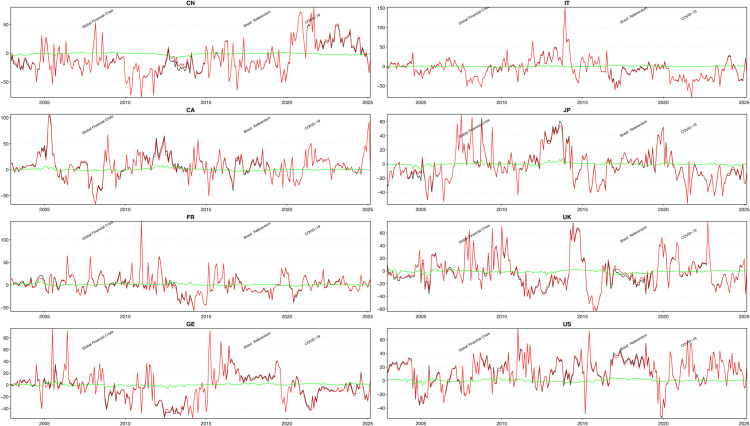
Net Dynamic Total Directional Connectedness: Short-term, Long-term and Overall.

**Fig 4:**
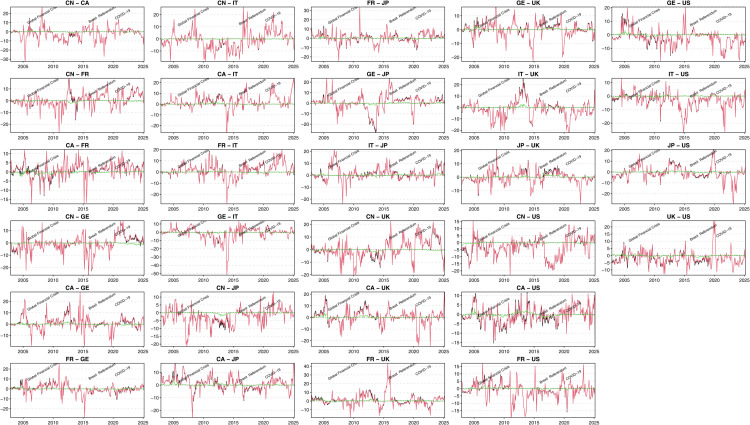
Dynamic Net Pairwise Directional Connectedness: Short-term, Long-term, and Overall.

**Fig 5:**
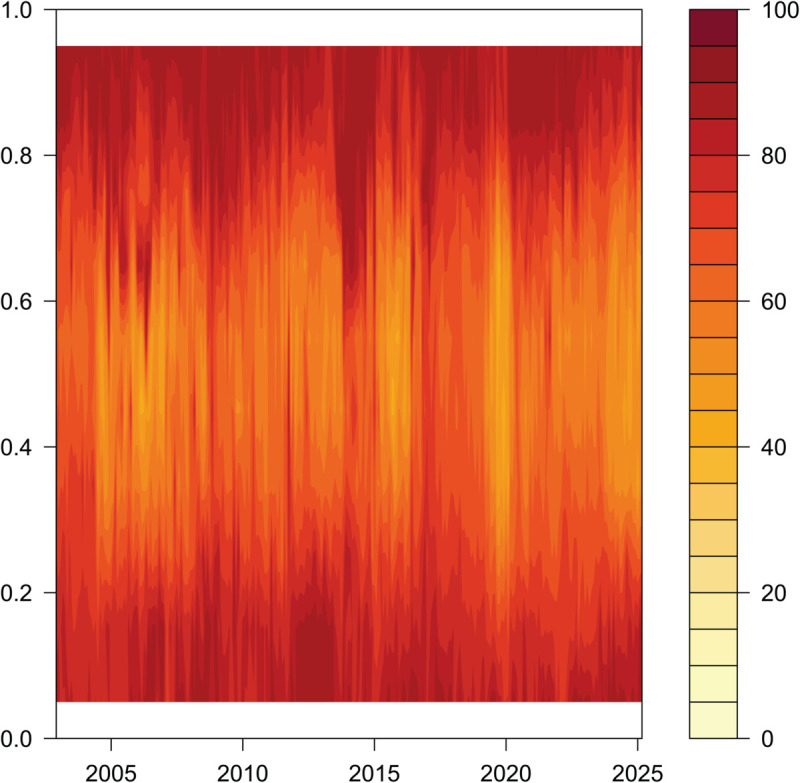
Overall Dynamic Total Connectedness in the Context of Time and Quantiles.

**Fig 6:**
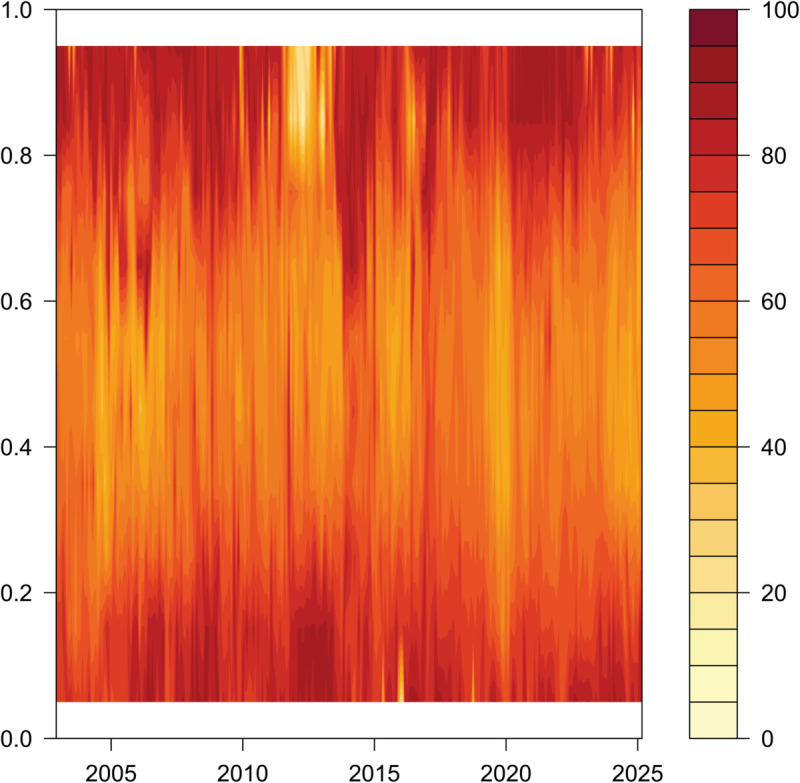
Short - term Dynamic Total Connectedness in the Context of Time and Quantiles.

**Fig 7:**
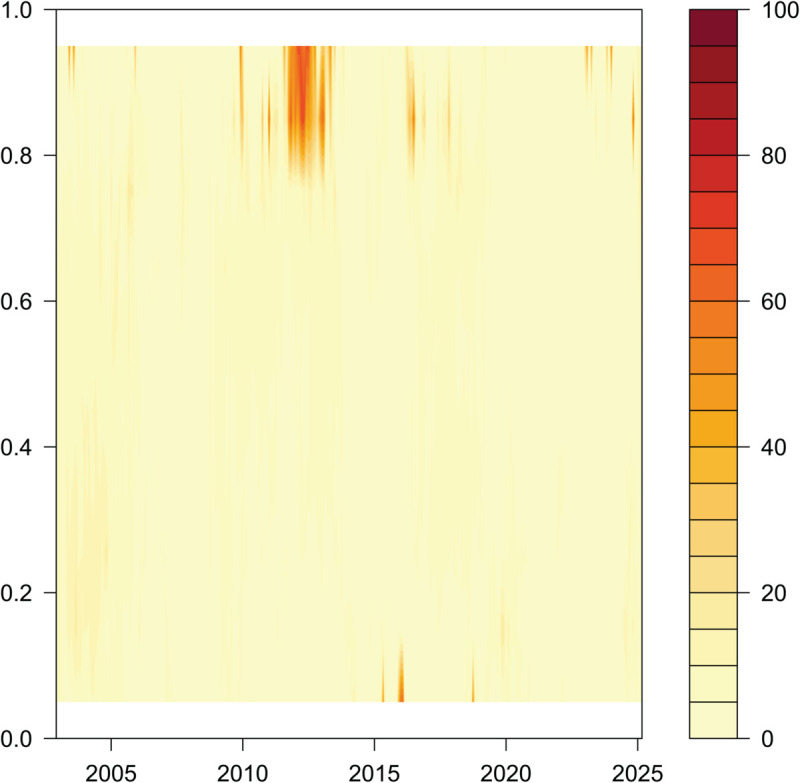
Long - term Dynamic Total Connectedness in the Context of Time and Quantiles.

**Table 3:** Averaged static connectedness.

	CN	CA	FR	GE	IT	JP	UK	US	FROM
*CN*	43.1	8.42	7.31	8.07	7.35	6.64	8.24	10.88	56.9
	(41.63,1.47)	(8.18,0.23)	(6.95,0.36)	(7.65,0.41)	(7.16,0.18)	(6.42,0.22)	(7.87,0.37)	(10.56,0.32)	(54.80,2.10)
*CA*	6.32	44.01	7.43	9.91	4.94	7.49	7.87	12.02	55.99
	(6.14,0.18)	(41.57,2.44)	(6.80,0.63)	(9.29,0.63)	(4.71,0.23)	(7.11,0.38)	(7.34,0.53)	(11.27,0.74)	(52.66,3.33)
*FR*	7.64	8.23	40.74	10.22	7.54	7.33	8.95	9.34	59.26
	(7.49,0.15)	(7.79,0.44)	(38.29,2.45)	(9.75,0.46)	(7.21,0.33)	(7.01,0.32)	(8.54,0.41)	(8.94,0.40)	(56.73,2.52)
*GE*	6.97	10.92	10.21	35.6	7.59	7.91	8.33	12.48	64.4
	(6.83,0.14)	(10.29,0.63)	(9.63,0.58)	(33.52,2.08)	(7.34,0.24)	(7.54,0.36)	(7.92,0.41)	(11.96,0.52)	(61.51,2.88)
*IT*	6.59	7.46	9.38	6.34	45.35	6.76	8.63	9.5	54.65
	(6.47,0.12)	(7.12,0.34)	(9.10,0.28)	(6.12,0.22)	(43.25,2.10)	(6.51,0.25)	(8.34,0.29)	(9.14,0.35)	(52.81,1.84)
*JP*	4.18	8.88	8.18	8.3	8.15	45.74	6.32	10.26	54.26
	(4.03,0.15)	(8.40,0.47)	(7.72,0.46)	(7.89,0.41)	(7.76,0.39)	(42.71,3.03)	(6.12,0.19)	(9.66,0.60)	(51.58,2.68)
*UK*	8.24	8.55	10.29	8.41	7.18	6.41	41.53	9.39	58.47
	(8.16,0.08)	(7.83,0.73)	(9.54,0.75)	(7.83,0.58)	(6.69,0.48)	(5.96,0.45)	(38.87,2.66)	(8.82,0.57)	(54.84,3.63)
*US*	7.5	11.41	8.18	10.28	6.2	9.38	7.52	39.53	60.47
	(7.33,0.17)	(10.80,0.61)	(7.73,0.45)	(9.72,0.55)	(5.87,0.34)	(8.78,0.60)	(7.04,0.48)	(37.30,2.23)	(57.27,3.20)
*TO*	47.44	63.86	60.98	61.53	48.95	51.92	55.86	73.86	TCI
	(46.45, 0.99)	(60.40, 3.45)	(57.47, 3.51)	(58.26, 3.27)	(46.75, 2.20)	(49.34, 2.58)	(53.17, 2.69)	(70.35, 3.51)	
*NET*	–9.46	7.87	1.72	–2.87	–5.7	–2.34	–2.61	13.39	58.08
	(–8.35,–1.11)	(7.74, 0.13)	(0.74, 0.99)	(–3.25, 0.38)	(–6.06, 0.36)	(–2.24,–0.11)	(–1.66,–0.95)	(13.08, 0.31)	(55.28,2.77)

Note: The results are derived from a 36-month rolling window QVAR model with a lag order of 1 (BIC) and a 20-step ahead generalized forecast error variance decomposition. The values in parentheses represent frequency-domain measures of connectedness, with the first and second entries corresponding to short- and long-term horizons, respectively. The remaining values represent time-domain connectedness measures across corresponding time horizons.

## Empirical results

### Research hypothesis

In the context of globalization, developed economies generally tend to strengthen the transmission of EPU through trade and investment, relying on their mature financial market systems, extensive international trade networks, and leading positions in international economic governance. In other words, the more developed the economy, the greater its influence on the development of other economies and the stronger the spillover effect of EPU. Based on the above analysis, this paper proposes the first hypothesis:

**Hypothesis 1.** In general, there is a significant positive correlation between the level of economic development and the strength of its EPU spillover effect.

The strength of EPU connectedness can be related to its fluctuation value. When the fluctuation value of EPU is at a high level, it means that the economic operation faces more potential risks and uncertainties. At this time, if the economy encounters external shocks, whether it is turbulence in the international financial market, aggravation of trade frictions, or geopolitical conflicts, it will easily cause the economy to send signals to the outside world that future economic uncertainty is increasing. This signaling mechanism will trigger a chain reaction in the global economic network, significantly increasing the connectedness of the EPU between that economy and other economies. This phenomenon is similar to the research conclusions of Bernanke and Kuttner [36], who pointed out that market reactions are biased towards changes in monetary policy rather than the state. Based on the above analysis, this paper proposes a second hypothesis:

**Hypothesis 2.** The more drastic the changes in EPU, the stronger the EPU connectedness between economies.

### Static connectedness in different frequency domains under the median condition

Table 3 reports the EPU connectedness between the sample countries under the median condition, with different frequencies (short-term and long-term) and aggregated. In Table 3, first, the last column (FROM) is the connectedness of each country affected by EPU shocks from other countries, that is, the receiving effect of external shocks. The value is the sum of each element in each row except the diagonal line; second, the penultimate row (TO) reflects each country’s connectedness to shocks from other countries, i.e., the sending effect of external shocks, and the value is the sum of each element in each column except the diagonal line; third, the penultimate row (NET) represents the net level of connectedness of each country, where a positive value indicates that the country is a net sender and a negative value indicates a net receiver; finally, TCI represents the total level of connectedness, which is calculated by dividing the sum of the FROM column or the TO row by the total number of sample countries N (N is 8 in this paper). From Table 3, we can observe the following phenomena:

1. From the TCI average, the total connectedness index is 58.08%, which indicates that the connectedness among the sample countries through EPU is relatively strong, and the spillover effect among them is obvious. The short-term connectedness level is 55.28%, which is about 20 times higher than the long-term connectedness level (2.77%). Based on static analysis, the total connectedness index is mainly contributed by the United States (73.86%), Canada (63.86%), Germany (61.53%) and France (60.98%).
2. There is heterogeneity in EPU connectedness among the sample economies. In the short and long run, the EPU reception levels of the eight economies range from the lowest in the short run in Japan (51.58%) to the highest in Germany (61.51%); the lowest in the long run in Italy (1.84%), followed by China (2.10%), and the highest in the United Kingdom (3.63%). Accordingly, the values of the short-term spillover level range from 46.45% in China to 70.35% in the United States, and the long-term spillover level ranges from 0.99% in China to 3.51% in the United States.
3. Japan’s EPU has the highest self-variance spillover of 45.74%, of which 42.71% comes from the short-term connectedness and 3.03% from the long-term connectedness. The EPU of other sample countries bears 54.26% of the forecast error variance of Japan’s EPU, that is, the total spillover from Japan’s EPU to other countries is 54.26%, while the total spillover to other countries is 51.92%. Therefore, its total net connectedness level can be calculated as -2.34%.
4. The spillover and reception levels of EPU in the sample countries do not always show a positive correlation, i.e., a higher reception level does not necessarily correspond to a higher spillover level, which leads to an inconsistent ranking of spillover and reception levels in the sample countries.
5. In terms of net receivers/senders, other countries except Germany and Italy show the same role in the short and long term, i.e., either net receivers or net senders. China is the largest net receiver (–9.46%), and this result is consistent in both the short and long term.
6. From a frequency domain perspective, regarding static connectivity under median conditions, within the sample period, the long-term connectivity among sample countries is significantly lower than their short-term connectivity. That is, a substantial portion of the overall effect of EPU contagion within sample countries occurs in the short term, while such interconnectedness remains very limited in the long term.

The above confirms the validity of Hypothesis 1 in this paper.

### Dynamic connectedness in different frequency domains under median conditions

The static analysis in the previous section only revealed the “average level” of connectedness during the sample period, and failed to thoroughly demonstrate its dynamic changes. This subsection further calculates the total connectedness, directed connectedness, and pairwise directed connectedness under the median condition, aiming to thoroughly analyze the dynamic evolution characteristics and inherent laws of EPU in the time domain among the sample countries.

#### Dynamic total connectedness under median conditions.

The total connectedness level reflects the overall magnitude of the EPU spillover effect among the sample countries, while the relative proportion of spillovers measures the extent to which changes in the EPU over different periods are caused by spillover effects. Fig 2 reports the dynamic evolution of the EPU’s connectedness. We can observe from Fig 2 that:

1. During the sample period, the overall dynamic TCI remained between 42% and 74%, indicating a strong connectedness between the EPUs of the sample countries. This connectedness effect has a significant impact on the first-order difference sequence of the EPU in both the short and long run.
2. The overall dynamic TCI and the short-term TCI show similar trends in the time dimension. In addition, the short-term TCI is significantly higher than the long-term TCI indicating that the short-term TCI has a more pronounced effect on the overall dynamic TCI. That is, during the sample period, changes in EPU were more influenced by short-term connectedness effects than by long-term connectedness effects. In other words, changes in overall TCI are mainly driven by short-term factors rather than long-term factors.
3. Long-term TCI is more stable. However, in the face of major crisis events, long-term TCI increases significantly. This phenomenon shows that major crisis events have a long-term negative impact on EPUs, which in turn leads to an increase in the long-term TCI between EPUs in the sample countries.
4. Different types of uncertainty shocks exert distinct impacts on connectivity, with the most pronounced effects observed during the 2008 global financial crisis and the COVID-19 pandemic. The 2008 crisis originated in the U.S. subprime mortgage market before spreading gradually worldwide, while the COVID-19 pandemic featured a near-simultaneous global outbreak. As a result, the increase in connectivity driven by the COVID-19 pandemic occurred far more rapidly than that driven by the 2008 crisis, with a also larger magnitude. In addition, due to lags in policy responses, the total TCI, short-term TCI, and long-term TCI within the sample period all plummeted to a relatively low level in the early phase of the COVID-19 outbreak (i.e., late 2019).

#### Net dynamic total directional connectedness under median conditions.

Fig 3 shows the results of the net dynamic total connectedness. A positive value indicates that the country is a net transmitter or sender of shocks, while a negative value means that it is a net receiver or recipient of shocks. The black, pink, and green lines in the figure represent net total connectedness, short-term net connectedness, and long-term net connectedness, respectively. The following phenomena can be clearly observed from Fig 3:

1. During the sample period, short-term net connectedness played a dominant role in explaining changes in the level of total net connectedness and showed a strong co-directional fluctuation with it. This suggests that short-term connectedness has a strong explanatory power for total net connectedness.
2. In almost all countries in the sample, the short-term volatility of the EPU is significantly higher than the long-term volatility of the EPU. This indicates that short-term economic policies are more vulnerable to external shocks and thus exhibit greater volatility, while long-term policies have relatively stable coping mechanisms and exhibit relatively low volatility.
3. In the short run, China was a net recipient for most of the sample period, and the range of the recipient level was significantly larger than that of the spillover level. In contrast, the United States was a net spiller, and the range of the spillover level was larger than that of the receiver level. Before the outbreak of the COVID-19 pandemic, China was almost always a net receiver of shocks, but after the outbreak it became a net transmitter for most of the time. This change reflects China’s changing role and adaptation to the drastic changes in the external environment.
4. In the long run, the fluctuations in the long-term net connectedness among the sample countries’ EPU are significantly lower than those in the short run. This implies that although the sample countries may experience greater fluctuations in the short run when faced with external shocks, they tend to respond with proactive policies to maintain long-term economic policy stability. This also shows that the sample countries are more inclined to respond to shocks by adjusting their policy frameworks in the long run, thereby reducing the persistent impact of uncertainty on the economy.

In summary, Fig 3 reflects the characteristics of the net connectedness of the sample countries in the face of external shocks and its changes over time. There is a significant difference between short-term and long-term connectedness. Short-term fluctuations reflect the sample countries’ rapid response to short-term uncertainties, while long-term stability shows the sample countries’ policy resilience and adaptability.

#### Net dynamic pairwise directional connectedness.

Fig 4 shows the net dynamic paired directional connectedness. The following phenomena can be observed in this figure:

1. For most of the sample period, the connectedness between the EPUs of the sample countries was not very evident. This shows that, under normal circumstances, the sample countries have only weak links with each other. Only when facing major shocks from economic, financial or political events did the sample countries’ EPUs show significant mutual transmission effects.
2. The dynamic changes in the overall net pairwise connectedness index between the sample countries’ EPUs are mainly driven by short-term connectedness fluctuations, which means that the impact of short-term fluctuations on the connectedness between the sample countries’ EPUs is more obvious.
3. The long-term net pairwise connectedness among EPUs in the sample countries is relatively smooth, while the level of long-term net pairwise connectedness does not necessarily increase sharply in the face of a major crisis event shock. This suggests that fluctuations in long-term connectedness are the result of multiple factors within the networked system of EPU rather than a sudden effect caused by a single event shock.
4. China’s EPU connectedness with the G7 countries is characterized by both overall and short-term pairwise connectedness. However, in terms of overall long-term pairwise connectedness, China’s influence is limited, both as a net recipient and as a net sender. This suggests that China’s role in long-term EPU transmission is relatively weak and does not significantly change the overall connectedness structure.

### Dynamic connectedness under different quantile conditions

Figs 5, 6, and 7 report the dynamic characteristics of overall, short-run, and long-run total connectedness under different quantile conditions of EPU in China and the G7 countries. In Figs 5, 6, and 7, the shading in the vertical direction represents the strength of connectedness under different quantile conditions.

#### Total dynamic connectedness under time domain quantile conditions (Fig 5).

1. Impact of Quantile Differences on Connectivity. Quantile levels intuitively reflect the degree of uncertainty. When in lower or higher quantile intervals, the connectivity of EPU remains at a relatively high level, indicating that “extreme uncertainty” significantly enhances the linkage and transmission effects of EPU between countries. In contrast, in the middle quantile intervals (such as the “conventional uncertainty interval” near the mean on the vertical axis), EPU connectivity is roughly symmetrically distributed, with relatively stable and consistent levels, suggesting that under conventional uncertainty conditions, cross-country EPU connectivity exhibits symmetry and stability without significant bias.
2. Differences in Outcomes Under Different Types of Uncertainty Shocks. For example, two distinct types of major uncertainty shocks—the 2008 global financial crisis and the 2020 COVID-19 pandemic—both led to a significant enhancement in EPU connectivity across different quantiles, but with clear heterogeneity. The 2008 global financial crisis, as a “systemic financial shock,” had widespread and profound impacts on the global economic and financial system. During the outbreak, spread, and subsequent adjustment phases, this shock exerted a “persistent strengthening effect” on connectivity across all quantiles, particularly driving connectivity to a peak at the highest quantile. The duration and depth of its impact significantly exceeded those of general event shocks, highlighting the long-term shaping ability of systemic crises on cross-country EPU linkages. The COVID-19 shock, due to its “suddenness and global nature,” led to a sudden increase in the EPU indices of all sample countries (Fig 1). Compared to normal periods, this crisis brought about an “explosive enhancement” of connectivity across all quantiles within a short time.

In summary, the total dynamic connectivity of EPU between China and the G7 countries is more pronounced under lower and higher quantile conditions, with enhanced linkages under major event shocks, while exhibiting stable and symmetric characteristics under non-extreme conditions. At the same time, the impact of different types of uncertainty shocks on connectivity shows significant heterogeneity.

#### Short-term dynamic TCI under the time domain quantile condition (Fig 6).

1. Impact of Quantile Differences on Connectedness. From the perspective of quantile differences, the short-term total connectedness among sample countries exhibits significant heterogeneity across various quantile levels. Under middle and low quantile conditions, the cross-border connectedness of Economic Policy Uncertainty (EPU) remains relatively stable and moderate, suggesting that the international transmission of uncertainty under normal circumstances is limited in intensity. However, as the quantile level rises to the upper tail, the degree of connectedness increases markedly, indicating a tighter cross-border linkage of EPU in scenarios characterized by heightened uncertainty. Moreover, the connectedness across quantiles displays an asymmetric pattern. In particular, when EPU experiences positive shocks, the connectedness effect becomes more pronounced, implying that positive shocks tend to have a stronger cross-border impact than negative ones. Nevertheless, in certain cases—such as during the early 2010s, a period marked by regional or policy-specific uncertainties—the connectedness under high quantile conditions declined. This reflects the nonlinear adjustment mechanism of EPU linkages under extreme conditions in the short term.
2. Differential Impacts of Various Types of Uncertainty Shocks. As illustrated by the heat map in Fig 6, the short-term connectedness of Economic Policy Uncertainty (EPU) between China and G7 countries during the 2008 global financial crisis and the COVID-19 pandemic exhibits notable differences in both pattern and persistence. During the 2008 financial crisis, driven by the contagion of systemic financial risks, the short-term connectedness of EPU—reflected by the intensity of color in the figure—rose sharply. This surge was concentrated in the initial outbreak phase of the crisis. However, as financial systems stabilized and the crisis subsided, the high level of connectedness was relatively short-lived, followed by a gradual decline. This suggests that uncertainty shocks originating in the financial sector tend to follow a clear “crisis outbreak – order restoration” trajectory in terms of their cross-border impact. In contrast, the COVID-19 pandemic, as a public health emergency, had a more global, persistent, and multi-dimensional impact. The economic shutdowns triggered by the pandemic, along with prolonged and complex policy responses—such as public health interventions and large-scale fiscal and monetary stimuli—led to a rapid and sustained increase in EPU connectedness around 2020. The persistence of this high level of connectedness is clearly visible in the figure, as indicated by the continuity of the dark-colored regions during this period.

In summary, although both shocks represent positive EPU shocks, the pandemic-related shock spans a broader range of domains and involves more enduring policy responses. Consequently, its impact on short-term connectedness is characterized by both a significant increase in intensity and prolonged persistence. In contrast, the financial crisis shock is marked by a rapid surge in connectedness followed by a relatively swift decline.

#### Long-term dynamic TCI under the time domain quantile condition (Fig 7).

1. Impact of Quantile Differences on Connectedness. Compared with its impact on short-term systemic connectivity, EPU’s influence on long-term systemic connectivity is negligible across all quantiles over the entire sample period. Moreover, long-term connectivity not only exhibits the same significant quantile heterogeneity as its short-term counterpart, but also responds more strongly to positive uncertainty shocks than to negative ones. Specifically, as shown in Fig 7, the effect on long-term total connectivity is significantly stronger when EPU falls within the higher quantile range (i.e., periods of large positive EPU changes) than during its lower quantile range (i.e., periods of large negative EPU changes). Visually, in terms of color, areas corresponding to higher EPU quantiles tend to be dark colors (e.g., orange-red)—a pattern that reflects higher intensity and coverage of long-term connectivity. In contrast, areas for lower EPU quantiles are mostly light yellow, indicating relatively weaker long-term connectivity. These observations underscore the nonlinearity and asymmetry of uncertainty shocks.
2. During the sample period, although exogenous shocks—such as the 2008 global financial crisis, Brexit, trade frictions, and the COVID-19 pandemic—differed significantly in their sources and transmission channels, none exhibited the “explosive” and “pulse-like” characteristics of short-term connectivity, nor did they exert a persistent impact on long-term connectivity. The European debt crisis, however, stands as an exception.

In summary, the connectedness effect between EPUs is stronger in extreme states compared to the normal state. At the same time, both the short-run and the long-run total connectedness indices show asymmetric characteristics. This suggests that the connectedness patterns among EPU in the sample countries differ in the face of external shocks, and that in extreme states, the short- and long-term connectedness effects do not manifest in the same way. At the same time, it also confirms the validity of Hypothesis 2 of this paper.

### Robust test

**Fig 8:**
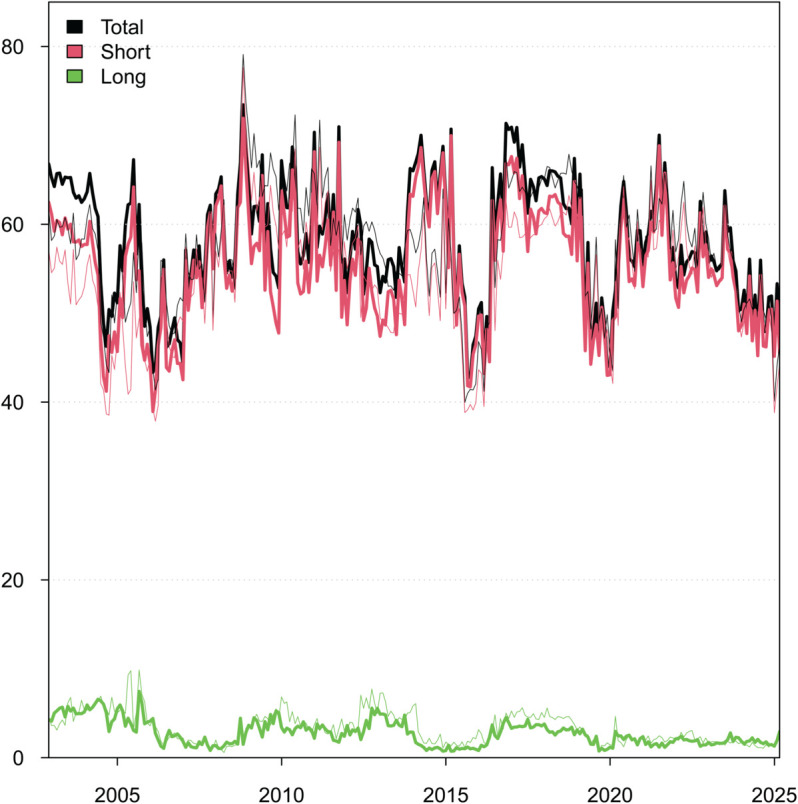
Dynamics of Total Connectedness: Short-Term, Long-Term, and Overall.

To verify the robustness of the results, we replaced the Chinese data used above with the China EPU index constructed by Huang and Luk [11] and recalculated it to obtain the robustness test results, as shown in Fig 8. In Fig 8, the thick line represents the results based on the data used above, while the thin line represents the results calculated using the data from Huang and Luk [11]. These results are all based on a QVAR model that uses a 36-month rolling window, a first-order lag length, and a generalized forecast error decomposition with 20 forward steps.

## Conclusion

Based on the analytical framework of a frequency-domain quantile econometric model, this paper systematically analyzes the connectivity of EPU between China and the G7 countries from January 2000 to January 2025 and its transmission mechanisms. The study reveals that the EPU connectivity between China and the G7 countries exhibits three key characteristics:

First, the connectivity shows significant asymmetry. In terms of the median level of connectivity, the United States occupies a dominant position in systemic spillovers in both the short and long term, with its spillover intensity significantly higher than that of other G7 economies. In contrast, China consistently acts as a net receiver of EPU shocks. Divergence is also observed within the G7: Germany and Italy exhibit significant heterogeneity in both short-term and long-term total connectivity, while the remaining economies tend to converge.

Second, short-term dynamic factors dominate the overall connectivity. The contribution of short-term factors to the net connectivity of the EPU network far exceeds that of long-term factors. The overall connectivity among the sample countries is primarily driven by short-term Total Connectivity Indices (TCIs), with the influence of long-term trends being relatively limited.

Third, connectivity significantly intensifies under extreme market conditions. The dynamic total connectivity demonstrates particularly prominent strength during extreme market states (e.g., crisis periods), indicating that the cross-border transmission of EPU is further amplified during risk accumulation phases.

Unlike the existing literature, which often focuses solely on the “mean level” (overlooking differences in extreme states) or a “single frequency” (failing to distinguish between short-term fluctuations and long-term trends), this study, by employing a frequency-domain quantile model, simultaneously incorporates both the “quantile dimension” (capturing differences between extreme and normal states) and the “frequency dimension” (distinguishing short-term from long-term linkages) for the first time in academic research. This approach clearly reveals the “conditional” (stronger transmission during extreme states) and “periodic” (short-term transmission dominates) characteristics of EPU spillover effects, thereby filling a research gap in the transmission mechanisms of international policy uncertainty across economies concerning the dual dimensions of “extreme scenarios—multiple time scales.”

The findings above carry clear policy implications for the G7 nations, China, and global financial stability governance. Firstly, as the United States is the primary source of global EPU spillovers, its economic policymaking should enhance “awareness of cross-border spillovers.” It should avoid frequent policy adjustments or poor expectation management that could lead to the global propagation of uncertainty shocks, which is a crucial prerequisite for maintaining global financial market stability. Secondly, as a net receiver of EPU, China should establish a dual response mechanism of “short-term hedging + long-term resilience.” In the short term, it can buffer external shocks by improving macroprudential policy tools (such as managing cross-border capital flows and guiding market expectations). In the long run, it should strengthen its endogenous economic growth drivers (e.g., promoting industrial upgrading and expanding domestic demand) to reduce sensitivity to external EPU. Thirdly, the heterogeneity observed in the connectivity of Germany and Italy suggests that policy coordination among G7 countries needs to move beyond the “homogeneity assumption” and fully consider the differences in transmission characteristics among economies to improve the effectiveness of policy coordination. Finally, given that short-term linkages dominate the overall connectivity and intensify significantly under extreme conditions, policymakers in various countries need to establish a collaborative mechanism prioritizing “short-term response and extreme risk early warning.” For instance, central banks could enhance response efficiency to short-term shocks through strengthened cross-border policy communication and develop contingency plans for extreme scenarios to mitigate the amplification effects of uncertainty transmission on global financial markets, thereby providing institutional safeguards for maintaining financial stability.

In summary, through the unique perspective of a frequency-domain quantile model, this study not only reveals the static characteristics and dynamic evolution of EPU connectivity between China and the G7 countries but also provides more precise empirical evidence on the transmission mechanisms of international policy uncertainty via the dual-dimensional analysis of “quantile-frequency.” The conclusions offer significant reference value for improving the global governance system for uncertainty risks.
